# 

**Published:** 1883-01

**Authors:** 


					CONTENTS
Aht. T.?
Drs. Watt and Taf< on the First P m mcni M ^lar*
.385
Abt. II -
-C.iiie- a^d Nmosis of the Max .larv Bon s.
By Truman Bmpliv M L).. D D. S.
.31)5
Akt. TIT.
r-Eii"lwffV of Dental Canes
By Geo, W>it?. M. D., D. D. S.
.405
Art. IV.
?A CI iss <>? Pulpites Tee.li, their TieiMnent.
By Dr. A. M R ss
409
Art V.
C<>mpiess?'?l Ninons Oxide.
By A.1 red Coleman. L. D S? E c.
413
Akt. VI.
-Dental FMuR
Bv Chevalier Cacciaourra. M. D.
417
Akt. VII
-Rem 'V tl of L< f< Superior mid Ciie< k >oi Sarcoma.
Bv Geo. L-twsnn F R. C. S
419
Art. VIII
Fracture of Upper Molar by.a Fall.
Bv C Vim eni Cotier. 11 L. .1). 8.
.421
Akt. IX ?
?What th<- Cd) Can D<>.
By Pro*. P Cohn
.452
Dental Department of University of California..
423
EDITORIAL, ETC.
Liability of Dentists.
424
A Gtneious C?uMihutinn 'o thn Museum of I lie Dental Department ot tlie
University of Manlmd..
425
Withdrawal of Dr. Jamea B. Hodgkin.
425
OBITUARY, ETC.
Dr. Mart-hall H. Webb.
420
BIBLIOGRAPHICAL NOTICES.
Tafts Practical Treat'ies on Operative Dentistry
427
E'em'irsof Dent il Matcm am! Therap?Mitices.
428
The Ph umacopoeia ot the United Siatea of America,
428
MONTHLY SUMMARY.
Resolution of Alveolar Absce?s into Serous Cyst.
429
A New Remedy for Spasms.
430
Hem >rrhiiee aftfr Teeth Extraction.
431
Treatment of Diptheria.
.432

				

